# Vitamin C as a Potential Prophylactic Measure Against Frozen Shoulder in an In Vivo Shoulder Contracture Animal Model

**DOI:** 10.1177/03635465231172192

**Published:** 2023-05-30

**Authors:** Oscar Feusi, Thea Fleischmann, Conny Waschkies, Hans-Christoph Pape, Clément M.L. Werner, Simon Tiziani

**Affiliations:** † Department of Trauma, University Hospital Zurich, University of Zurich, Zurich, Switzerland; ‡ Division of Surgical Research, University Hospital Zurich, University of Zurich, Zurich, Switzerland; Investigation performed at University Hospital Zurich, University of Zurich, Zurich, Switzerland

**Keywords:** shoulder, magnetic resonance imaging, microscopic pathology, shoulder stiffness, frozen shoulder, vitamin C

## Abstract

**Background:**

Frozen shoulder is a common, painful, and movement-restricting condition. Although primary frozen shoulder is idiopathic, secondary frozen shoulder can occur after trauma or surgery. Prophylactic and therapeutic options are often unsatisfactory. Vitamin C (ascorbic acid) is a potent physiological antioxidant and likely inhibits the activation of nuclear factor κB, which plays a decisive role in inflammatory reactions.

**Hypothesis:**

Because of its anti-inflammatory effects, vitamin C may be valuable in the prevention of secondary frozen shoulder.

**Study Design:**

Controlled laboratory study.

**Methods:**

An in vivo shoulder contracture model was conducted by fixation of the right proximal limb of Sprague-Dawley rats. A treatment group (n = 8) receiving vitamin C orally was compared with a control group (n = 9) without vitamin C. The primary outcome was capsular thickness at the shoulder joint measured on magnetic resonance imaging (MRI) examination. Further histological examination was performed but was not statistically analyzed because of variability of the cutting plane through the glenoid.

**Results:**

Vitamin C treatment resulted in less thickening of the axillary fold of the operated shoulder at 2 of the 3 locations measured on MRI compared with untreated controls (insertion to the glenoid, *P* = .074; insertion to the humerus, *P* = .006; middle of the axillary recess, *P* = .008). The observed structural changes in histological examination corroborated the significant changes obtained from the MRI measurements.

**Conclusion:**

Prophylactic vitamin C seemed to reduce the thickening of the axillary recess in secondary frozen shoulder in this preclinical study.

**Clinical Relevance:**

Vitamin C may be helpful as a noninvasive therapeutic measure to prevent secondary frozen shoulder (eg, within the context of surgery in the shoulder region or immobilization) or to treat primary frozen shoulder at an early stage. Further studies are required to evaluate the effect of this treatment in humans and the necessary dosage in humans.

Frozen shoulder is a musculoskeletal condition that can manifest clinically with a slow onset of pain close to the insertion of the deltoid muscle, often leading to sleep problems. Further, elevation and external rotation are restricted due to pain, without presenting any relevant changes on a conventional radiograph.^2,9,14^ Three stages are described: the painful “freezing” phase, with night pain and progressive active and passive stiffness; the “frozen” phase, in which the pain slowly decreases; and a “thawing” phase that entails spontaneous improvement of shoulder function.^35^ The average duration until recovery, which often is not complete, is about 30 months but can extend to >3 years.^22,39^

Frozen shoulder has its peak incidence in patients between the ages of 40 and 60 years^42^ and affects around 2% of the population.^5^ Although primary frozen shoulder is idiopathic, secondary frozen shoulder can occur after trauma (eg, fractures) and surgery or can accompany impingement, calcific tendinitis, or arthritis.^8,42^ Postoperative secondary frozen shoulder is an important complication of surgical repair of the rotator cuff.^17,23^ Another potential cause is immobilization,^22^ which may induce inflammation,^48^ and contraction^47^ of the glenohumeral joint.

Treatment options such as nonsteroidal anti-inflammatory drugs, physical therapy, steroid therapy, distension arthrography, therapeutic ultrasound, manipulation under anesthesia, arthroscopic capsular release, and open surgical release offer little long-term advantage and are controversially discussed in the literature.^6,7,16,19,21,40-42,44^ Furthermore, treatment options need to be distinguished from prophylactic measures that may be applied postoperatively (eg, after arthroscopic rotator cuff repair) or when long-term immobilization is expected (eg, sparing in an orthosis). Therefore, it is advisable to conduct further studies in search of effective prevention and treatment of frozen shoulder.

Vitamin C (ascorbic acid) is a potent physiological antioxidant that protects tissue from harmful oxidative stress.^4,12^ Vitamin C likely inhibits the activation of the nuclear transcription factor κB (NF-κB), which regulates the expression of a variety of inflammation-relevant genes and thus plays a decisive role in inflammatory reactions.^1,3,4,11^ The inhibition of NF-κB by vitamin C is not only an antioxidant effect, because activation of redox-insensitive pathways is prevented as well.^4^

We therefore hypothesized that vitamin C may be valuable in the prevention of secondary frozen shoulder due to its anti-inflammatory effects by inhibition of NF-κB.

In this study, we used an established in vivo contracture model in rats^18,27,36,37^ to test the prophylactic effect of vitamin C in secondary frozen shoulder.

## Methods

### Ethics Statement

Animal housing and all procedures and protocols were approved by the Cantonal Veterinary Office, Zurich, Switzerland (license No. ZH139/17). The study was performed in accordance with Swiss animal protection law and conformed to European Directive 2010/63/EU of the European Parliament and of the Council of 22 September 2010 on the Protection of Animals Used for Scientific Purposes and the Guide for the Care and Use of Laboratory Animals.

### Study Design

We obtained 20 male Sprague-Dawley rats at the age of 3 months from a commercial supplier and housed them in groups of 2 or 3 animals under standardized conditions with ad libitum access to water and food in IVC type 1500 cages. After an acclimatization period of 1 week, the animals were randomly allocated into 2 groups of 10 rats each and underwent surgery immobilizing the right proximal limb to induce secondary frozen shoulder. Throughout the following 8 weeks, one group received high-dose vitamin C (Streuli Pharma AG) at a concentration of 10 g/L via drinking water so that a minimum daily intake of 100 mg per 100 g of body weight (BW) was guaranteed. The other 10 rats served as a control group and received no treatment. We measured water consumption of both groups on a daily basis by weighing the drinking bottles. The rats were sacrificed 8 weeks after surgery, and the harvested shoulders underwent magnetic resonance imaging (MRI) examination to measure capsular thickness at the shoulder joint. Further, histological examination was performed but was not statistically analyzed due to the variability of the cutting plane through the glenoid.

### Anesthesia and Preparation

We induced anesthesia in a sealed chamber with a mixture of 5% isoflurane and oxygen at a flow rate of approximately 600 mL/min and maintained it with 2% to 3% isoflurane and oxygen at a flow rate of 600 mL/min via a nose mask. After the disappearance of protective reflexes was verified, protective eye ointment was applied, and the right shoulder was shaved and disinfected. We provided analgesia by a subcutaneous injection of ketamine-hydrochloride (20 mg/kg BW) before surgery. Afterward, we placed the animal on a warming pad on the operating table. All rats were monitored during the procedure, and surgery was performed under aseptic conditions.

### Surgical Technique

The applied surgical technique was previously described by Ochiai et al,^36^ Kanno et al,^27^ and Oki et al.^37^ After positioning the animals in lateral recumbency, we palpated the surface landmarks on the right shoulder, including the spine of the scapula and the humerus. Surgical access occurred through a 1.5- to 2-cm incision over the lateral glenohumeral joint, followed by atraumatic preparation of the surface of the humerus. We passed a No. 1-0 FiberWire (Arthrex) with a curved surgical needle around the humerus and pulled it through the elevated facies costalis of the scapula without damaging the glenohumeral joint or structures of the thorax. Afterward, we attached the ends of this high-strength suture to each other, inducing the fixation of humerus and scapula in adduction and slight dorsal extension. The wound was closed with a skin suture using a No. 4-0 silk suture.

### Postoperative Procedures

So the animals could recover from anesthesia and to prevent harmful behavior among them, we kept the rats in single housing for the first hour after surgery and returned them afterward to their home cages. For postsurgical pain alleviation, subcutaneous injections of buprenorphine (0.1 mg/kg BW) were administered every 6 hours during the day. During the night, the rats had access to buprenorphine-medicated drinking water for up to 3 days after surgery. For the first 5 days after the operation, we monitored and scored the rats daily for locomotion, posture, general appearance, wound healing, social behavior, and weight loss, in order to detect any signs of pain or other complications. Afterward, monitoring was carried out once a week until the end of the experiment. The experiment ended 8 weeks after surgery by sacrificing the rats with carbon dioxide. Before harvesting the shoulders, we checked the persistence of the fixation by cautiously examining the decreased mobility in the operated joint. Both shoulders in each rat were removed extremely carefully to avoid inducing tensile force and thereby damaging the glenohumeral joint.

### Magnetic Resonance Imaging

The shoulders were dissected from the body without damaging the joint capsule and were subject to MRI analysis in a 4.7-T Bruker PharmaScan small animal MRI scanner (Bruker BioSpin). We took care to place the harvested shoulders in the MRI scanner in an orientation that was as consistent as possible. MRI was performed with a linearly polarized mouse volume resonator, and MRI scans were sampled in an oblique coronal plane through the glenoid of the scapula. All MRI scans from both shoulders were obtained with the following fat-suppressed MRI protocols: T1-weighted, high-resolution, fast low angle shot sequence (repetition time [TR]/echo time [TE], 425/6.9 ms; flip angle, 40°; 4 averages; spatial resolution, 100 × 100 µm^2^; slice thickness, 500 µm; slice gap, 100 µm) and a proton density/T2-weighted (PD/T2W) multispin-multiecho sequence (TR, 2000 ms; TE, 11.7/23.4/35.1 ms; 2 averages; spatial resolution, 150 × 150 µm^2^; slice thickness, 500 µm; slice gap, 100 µm).

We measured the thickness of the axillary recess (primary outcome) of both shoulders of the rats in the PD/T2W sequences right beside the insertion point of the capsule to the glenoid and to the humerus as well as in the middle of the axillary recess to minimize the effect of the variance of the cutting plane through the joint.

### Histopathological Examination

Histological examination was carried out on both the operated and the nonoperated shoulders of both groups. After decalcification of the samples with the decalcifying solution Usedecalc (Medite GmbH), two-thirds of the scapula and the humerus apart from the metaphysis as well as the superficial muscles were dissected from the shoulder, leaving the undamaged joint with as much surface as possible for the decalcification solution. We halved the samples through the glenoid and treated them again with the decalcifying solution. Afterward, the samples were dehydrated through formalin and ascending alcohol concentrations, defatted in xylene, waxed, and cast into paraffin blocks. The paraffin blocks were cut with a Leica RM2155 microtome (Leica Microsystems GmbH) into slides of 5-µm thickness representing the section through the glenoid. Last, we stained the samples with hematoxylin and eosin and trichrome using the hematoxylin solution according to Weigert (Carl Roth GmbH), ponceau acid fuchsin (Goldner solution I; Carl Roth GmbH), tungstophosphoric acid orange G (Goldner solution II; Carl Roth GmbH), acid green (Goldner solution III; Carl Roth GmbH), and acetic acid 100% (Merck KGaA).

### Statistical Analysis

Statistical analysis of the MRI data was performed using SPSS Statistics (Version 26 for Windows; IBM Corp). The Shapiro-Wilk test revealed nonnormal distribution of the data. To compare the outcome of the treatment group (vitamin C) with the outcome of the control group (no treatment), we used the Wilcoxon-Mann-Whitney 2-sample rank-sum test. The difference between the operated and the nonoperated shoulder within the rats was compared between groups. Further, each measured point was compared between the operated and the nonoperated side within the same specimen. *P* < .05 was considered significant.

## Results

The fixation in the right anterior limb manifested in a decrease of flexion, extension, abduction, and rotation, which was observed in all animals. Nevertheless, the general locomotion was not significantly limited, and all animals exhibited normal behavior (ie, eating and drinking, grooming, social interaction).

### Adverse Outcomes

One rat died during anesthesia at the beginning of surgery, and 2 rats in the treatment group had to be euthanized because of an abscess in the second week and the fifth week, respectively, leaving 9 animals in the control group and 8 animals in the treatment group. Two rats developed a wrist drop, probably due to surgical injury of the radial nerve that runs along the lateral humerus. It did not affect the behavior and general health of the animals. Three rats nibbled their sutures open and needed revision once on the day after surgery.

**Figure 1.**
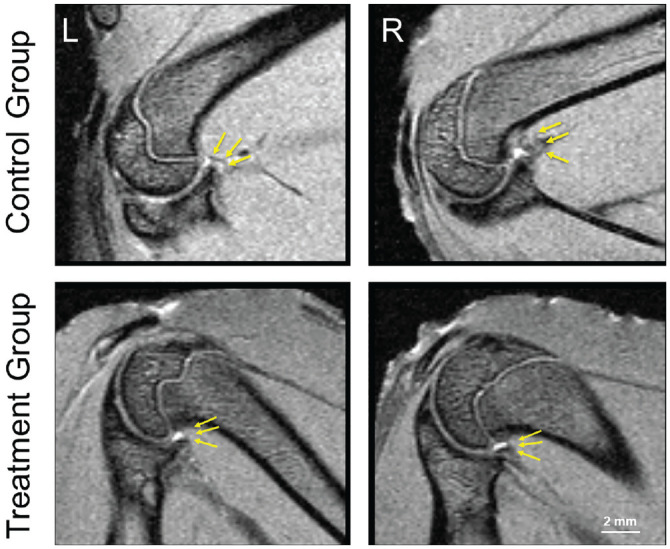
Magnetic resonance imaging scans (proton density/T2-weighted, multispin-multiecho sequence) of rat shoulders in the untreated control group (top row) and the treatment group (bottom row). L, left, nonoperated shoulder; R, right, operated shoulder. Arrows indicate measured locations. Thicknesses of the axillary recess were as follows. Nonoperated shoulder of the control group: insertion to glenoid, 0.450 mm; insertion to humerus, 0.480 mm; middle of axillary recess, 0.480 mm. Operated shoulder of the control group: insertion to glenoid, 0.640 mm; insertion to humerus, 1.090 mm; middle of axillary recess, 0.960 mm. Nonoperated shoulder of the treatment group: insertion to glenoid, 0.340 mm; insertion to humerus, 0.480 mm; middle of axillary recess, 0.480 mm. Operated shoulder of the treatment group: insertion to glenoid, 0.450 mm; insertion to humerus, 0.480 mm; middle of axillary recess, 0.480 mm.

Vitamin C treatment resulted in less thickening of the axillary fold of the operated shoulder at 2 of the 3 locations measured on MRI, compared with untreated controls. Figure 1 shows representative MRI scans of 1 rat of each group illustrating the quantification strategy. Table 1 provides the measurement results.

**Table 1:** Thickness of Axillary Recess^*a*^

Measurement Location	Group	Nonoperated Shoulder (left)	Operated Shoulder (right)
Insertion to glenoid	Control	0.461 ± 0.056	0.723 ± 0.285
	Treatment (vitamin C)	0.533 ± 0.106	0.525 ± 0.118
Insertion to humerus	Control	0.551 ± 0.106	1.426 ± 0.498
	Treatment (vitamin C)	0.641 ± 0.133	0.826 ± 0.293
Middle of axillary recess	Control	0.516 ± 0.059	1.301 ± 0.561
	Treatment (vitamin C)	0.659 ± 0.240	0.766 ± 0.224

a Data are expressed in mm as mean ± SD.

**Figure 2.**
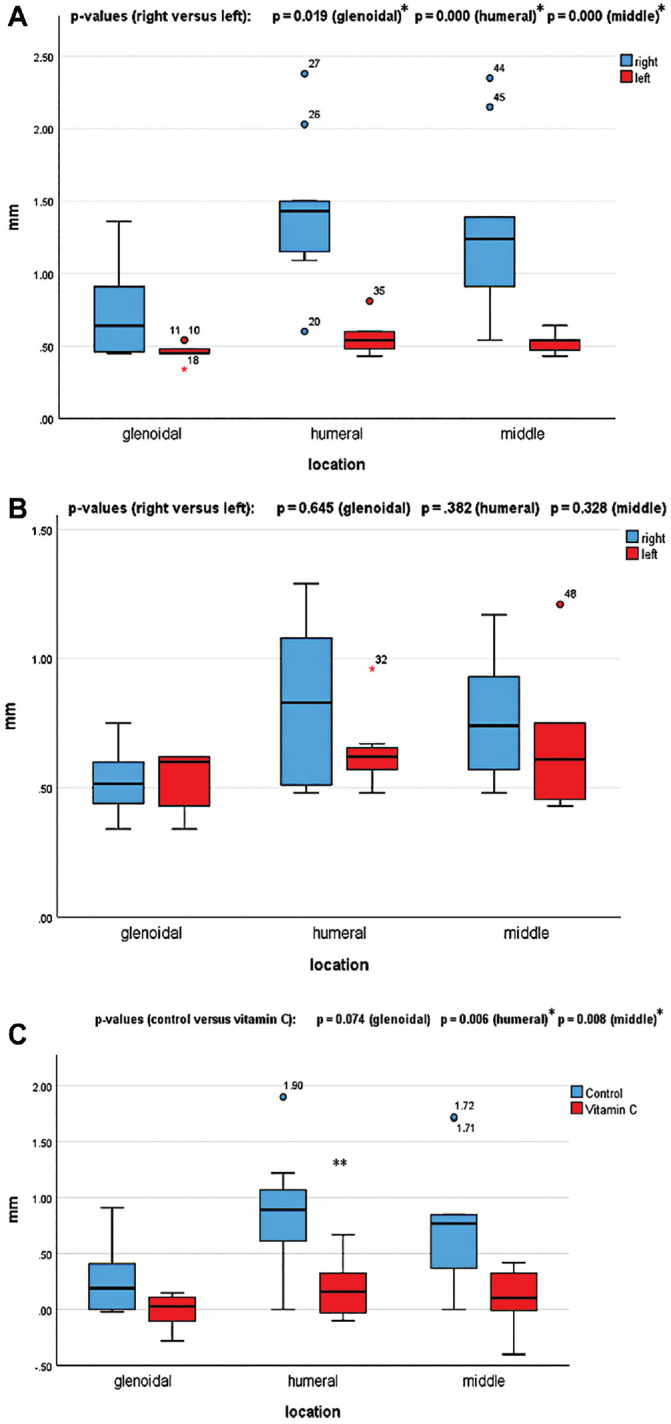
Boxplots of axillary recess thickness in (A) the untreated control group (n = 9) and (B) the treatment group (n = 8) at the 3 measured locations. *Right* refers to operated shoulders; *left* refers to nonoperated shoulders. (C) Boxplot of the difference in axillary recess thickness between the operated (right) and nonoperated (left) shoulders at the 3 measured locations for the control versus treatment groups (N =17). *Statistically significant; **outliers.

In the untreated control group, the axillary recess was significantly thicker in the operated shoulders (right) than in the nonoperated shoulders (left) at all 3 locations measured on MRI (insertion to glenoid, *P* = .019, effect size *r* = 0.569; insertion to humerus, *P* = .000, effect size *r* = 0.806; middle of axillary recess, *P* = .000, effect size *r* = 0.789) (Figure 2A).

In the treatment group, in none of the 3 locations measured on MRI was the axillary recess of the operated shoulders significantly thicker than in the nonoperated shoulders (insertion to glenoid, *P* = .645, effect size *r* = 0.120; insertion to humerus, *P* = .382, effect size *r* = 0.224; middle of axillary recess, *P* = .328, effect size *r* = 0.263) (Figure 2B).

The difference in axillary recess thickness between the operated and the nonoperated shoulder was significantly smaller in the treatment group than in the control group in 2 of the 3 locations measured on MRI (insertion to glenoid, *P* = .074, effect size *r* = 0.433; insertion to humerus, *P* = .006, effect size *r* = 0.654; middle of axillary recess, *P* = .008, effect size *r* = 0.619) (Figure 2C).

**Figure 3.**
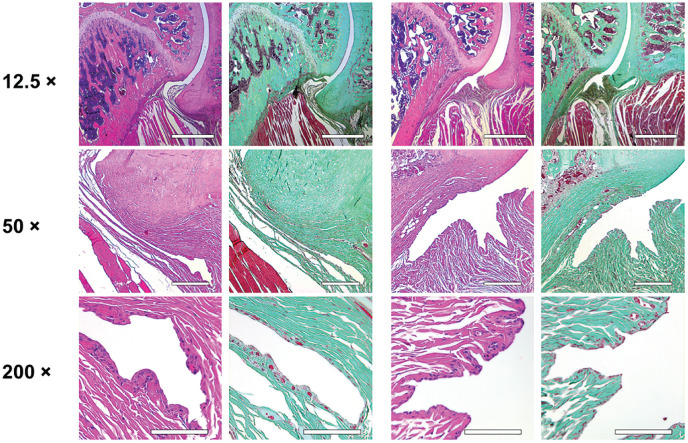
Histological examination of the nonoperated shoulders. Scale bar represents 500 µm in the ×12.5 magnification, 250 µm in the ×50 magnification, and 100 µm in the ×200 magnification. Control group (left 2 columns) and treatment group (right 2 columns): normal synovial folds with normal synovial cells, no fibrosis, and no hyperemia. For the control and treatment groups, hematoxylin and eosin staining is shown on the left and trichrome staining on the right.

**Figure 4.**
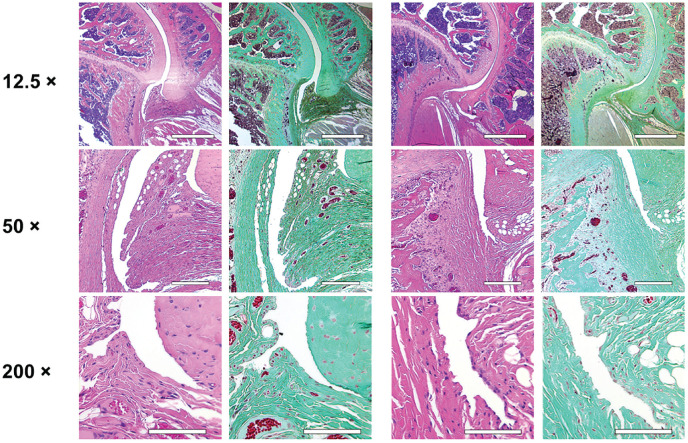
Histological examination of the operated shoulders. Scale bar represents 500 µm in the ×12.5 magnification, 250 µm in the ×50 magnification, and 100 µm in the ×200 magnification. Control group (left 2 columns): flattened synovial folds with loss or atrophy of synovial cells for the most part, fibrosis of the subsynovium and the fibrous capsule, hyperemia, and increase in vascularity. Treatment group (right 2 columns): flattened synovial folds with partial loss or atrophy of synovial cells, mild fibrosis of the subsynovium, and no hyperemia. For the control and treatment groups, hematoxylin and eosin staining is shown on the left and trichrome staining on the right.

Histological analysis of the affected shoulders in both groups presented the following structural changes in different grades of severity: hyperemia, atrophy of the synovial membrane, flattened synovial folds, fibrosis of the subsynovium, hyperemia, increased vascularity, and fibrosis in the fibrous capsule (Figures 3 and 4).

Due to variation of the cutting plane and because it was not possible to obtain an undamaged slide for every specimen given the artifacts caused by cutting through the complex structures of different tissue types, statistical analysis could not reliably be performed. However, the observed structural changes corroborate and explain the significant changes obtained from the MRI measurements.

All animals gained weight throughout the 8 weeks of the study period. At baseline, rats in the control group weighed between 256 and 288 g, whereas those in the treatment group weighed between 259 and 297 g. The weight of the rats at the end of the experiment ranged from 425 to 625 g with an average of 522.125 g in the treatment group (n = 8) and from 452 to 598 g with an average of 530.333 g in the control group (n = 9).

The treatment group drank more water than the control group. The rats of the control group drank on average 8.5 mL of water per 100 g BW per day. Table 2 provides the water and vitamin C intake of the treatment group.

**Table 2:** Average Consumption of Water Enriched With Vitamin C in the Treatment Group (n = 8)

Week	Water intake, mL^*a*^	Vitamin C, mg^*a*^
1	13.4	134
2	16.4	164
3	13.7	137
4	13.4	134
5	11.6	116
6	12.2	122
7	12.6	126
8	11.0	110

a per 100 g of body weight per day.

## Discussion

In this study, oral vitamin C reduced the thickening of the axillary recess in an animal model of secondary frozen shoulder. Surgical immobilization produced an increase in axillary fold thickness compared with the contralateral nonoperated shoulder in the untreated control group. The thickening in the control group was significantly more severe than in the vitamin C group in 2 of 3 measured parts of the joint. Only the measured locations at the insertion of the capsule to the glenoid revealed no significant differences between the 2 groups.

Besides thickening of the joint capsule, other factors have been described in the literature as possible MRI findings of frozen shoulder: a thickened coracohumeral ligament, obliteration of the subcoracoid fat triangle, and edema in the axillary recess.^13,30,38^ Mengiardi et al^31^ reported a significantly thicker joint capsule in the rotator cuff interval in patients with frozen shoulder, but not in the axillary recess, as well as a significantly thickened coracohumeral ligament and a significantly smaller volume of the axillary recess compared with a control group. Chi et al^13^ stated that frozen shoulder can be diagnosed on noncontrast MRI and presents with coracohumeral ligament thickening, rotator interval infiltration, and thickening and/or edema of the axillary recess. Park et al^38^ concluded that edema in the axillary recess is more typical for early stages, whereas there is more thickening of the joint capsule in the axillary recess in later stages of the condition.

Because the resolution of the obtained MRI scans, despite being obtained from a dedicated small animal MRI machine, is lower than the resolution in human shoulder imaging, the analysis in this study was limited to capsular thickness. We did not see any edema, possibly because contracture may be mature by 8 weeks or because of the limitations of small animal imaging.

The structural changes we saw in the histological analysis match the descriptions in other studies. The basic pathogenesis of frozen shoulder is still unknown. It is assumed that a painful inflammatory process precedes subsequent fibrosis entailing clinically visible stiffness.^10,20,33,42^ This can be observed histologically in the initial stage with hyperplastic synovial cells and sprouting of hyperemic vessels. After the inflammatory phase has subsided, the synovial membrane becomes atrophic, synovial folds flatten, type III collagen accumulates, and fibrosis predominates.^29,43^ Given the histologic features seen here, we believe that the 8-week interval most likely falls within the fibrotic phase.

Underlying a frozen shoulder, an inflammatory response to various trigger factors may lead to dysregulation of matrix metalloproteinases (MMPs) and tissue inhibitors of metalloproteinases (TIMPs) as well as increased expression of cytokines and growth factors such as interleukin 17A (IL-17A) and transforming growth factor β (TGF-β), which affect fibroblast growth and function and result in a pathological NF κB–dependent remodeling of the synovium and capsule.^1,24,26,33,44,45^

In an inflammatory process, oxidants activate MMPs and simultaneously deactivate their inhibitors (ie, TIMPs). MMPs degrade collagen and activate TGF-β, the most potent chemoattractant for neutrophils and a promoter of tissue repair. As a persistent stimulus, TGF-β leads to capsular fibrosis.^34,43^ Further, through increased IL-17A stimulation of T cells, fibroblasts produce greater fibrotic and inflammatory responses.^1,33^ Akbar et al^1^ prevented the profibrotic and inflammatory IL 17A–induced changes in frozen shoulder fibroblasts by inhibiting the NF-κB signaling pathway with antibodies. Vitamin C might interfere in this pathway by buffering the oxidants^4,12^ and by preventing the activation of NF-κB, which is a crucial regulator of inflammation.^1,3,4,11^

To the best of our knowledge, no literature is available regarding the use of vitamin C in frozen shoulder. This surprised us very much, especially because vitamin C in a dosage up to 1000 mg/d is already listed in patient brochures of several Swiss clinics as part of the treatment of frozen shoulder.^32^

Preclinical evidence indicates that vitamin C could be valuable in the treatment of musculoskeletal injuries. It accelerates bone healing after a fracture, increases type I collagen synthesis, and reduces oxidative stress parameters.^15^

We observed no clinical side effects due to vitamin C in this study. Vitamin C has a low toxicity. The most common complaints, such as diarrhea, nausea, abdominal cramps, and other gastrointestinal disorders, are caused by the osmotic effect of unabsorbed vitamin C when daily intake exceeds 2000 mg.^25^ Relevant side effects are not expected unless the dose exceeds the risk level for developing kidney stones or ulcers.^15^

The shoulder contracture model in this study was induced by soft tissue injury due to surgery and by immobilization, mimicking postoperative secondary frozen shoulder. To our knowledge, no model of an idiopathic frozen shoulder has been described.^27,29,36^ Although Rodeo et al^43^ found no significant differences between the primary (idiopathic) and the secondary form of frozen shoulder in regard to frequency and localization for cytokines, cells, and collagen types, Robinson et al^42^ reported an association with a poorer prognosis for the secondary form than for the idiopathic form. Therefore, the outcome of vitamin C treatment in the early stages of primary frozen shoulder might differ from that of the secondary form.

In orthopaedic research, large animals such as pigs, dogs, or sheep are often used with the advantage that the joints to be examined are robust and large and thus more accessible for investigation. However, this also entails a costly infrastructure. The use of smaller animal models such as mice or rats allows more economical research, but the structures to be studied are more fragile and the material available for studies is limited. Because of the pilot nature of the study, we chose a small animal model.

### Limitations

We did not measure joint range of motion or mechanical function, in order to prevent damaging the tissue before examination, which is the most distinct limitation of the study. Because vitamin C promotes collagen cross-linking, it is possible that despite a reduction in axillary thickness, there may be no difference in overall stiffness due to improved collagen organization and/or faster consolidation of the scar.

We did not investigate the necessary minimal concentrations or doses of vitamin C. Furthermore, a direct deduction of effective dosage for humans from our study is not possible due to the different metabolic turnovers in humans and rats.^28^ It is known that doses >100 mg/d lead to urinary excretion of unmetabolized ascorbate in human beings, and doses >500 mg/d do not increase vitamin C concentration in tissue.^49^

In contrast to humans, rats are capable of producing vitamin C by themselves via the enzyme L-gulonactone oxidase, which is necessary in the terminal pathway of vitamin C synthesis.^4^ This might appear as a limitation of our study; however, the rats received such high doses that the endogenous production (corresponding to approximately 3.91-19.86 mg per 100 g BW per day^28^) can probably be disregarded. Exchanging the rats with another species not capable of synthesizing vitamin C (eg, guinea pigs or nonhuman primates^30^) could not be justified through the effect on the results.

The treatment group (ie, animals receiving vitamin C via drinking water) drank on average more than the control group throughout the study phase. This also became apparent by more urine and soiling in the bedding, resulting in more frequent cage changes for the treatment group. We hypothesized that the rats liked the taste of the vitamin C solution more than plain water, but we did not consider the difference in the amount of water intake to be a confounder.

The number of animals in both groups was relatively small. However, we consider the sample size to be sufficient for the pilot nature of the study.

A conspicuous finding was that the axillary recesses of the nonoperated shoulders of the control group were on average 0.105 mm thinner than those in the treatment group. We have not found an explanation for this, but it should be mentioned because it can influence the outcome of the study and strengthens the difference between the left and right side in the control group. Nevertheless, the difference in the affected shoulders between the 2 groups is strongly significant.

Finally, there may be some differences between rat and human joints that limit the study, although the anatomy and function of rat shoulders are comparable with those of human shoulders (rat shoulder motion during forward walking resembles abduction of the human shoulder).^27,46^ Changes in gene expression pattern and histology described in frozen shoulder in humans have also been described in the shoulder contracture model used.^18,37^

## Conclusion

Prophylactic vitamin C seemed to reduce the thickening of the axillary recess in secondary frozen shoulder in rats. This was a preclinical study including MRI and histological analysis with a small number of rats. Vitamin C may be helpful as a noninvasive therapeutic measure in the prevention of secondary frozen shoulder (eg, within the context of surgery in the shoulder region or immobilization) or for treatment of primary frozen shoulder at an early stage. Further studies are required to evaluate the effect in humans and the necessary dosage in humans.
